# Fetal oxygenation in the last weeks of pregnancy evaluated through the umbilical cord blood gas analysis

**DOI:** 10.3389/fped.2023.1140021

**Published:** 2023-04-21

**Authors:** Luca Filippi, Rosa Teresa Scaramuzzo, Francesca Pascarella, Alessandro Pini, Riccardo Morganti, Maurizio Cammalleri, Paola Bagnoli, Massimiliano Ciantelli

**Affiliations:** ^1^ Department of Clinical and Experimental Medicine, University of Pisa, Pisa, Italy; ^2^ Neonatology Unit, Azienda Ospedaliero-Universitaria Pisana, Pisa, Italy; ^3^ Department of Experimental and Clinical Medicine, University of Florence, Florence, Italy; ^4^ Section of Statistics, Azienda Ospedaliero-Universitaria Pisana, Pisa, Italy; ^5^ Unit of General Physiology, Department of Biology, University of Pisa, Pisa, Italy

**Keywords:** newborn, intrauterine hypoxia, fetal hypoxia, warburg effect, differentiation

## Abstract

**Introduction:**

Embryo and fetus grow and mature over the first trimester of pregnancy in a dynamic hypoxic environment, where placenta development assures an increased oxygen availability. However, it is unclear whether and how oxygenation changes in the later trimesters and, more specifically, in the last weeks of pregnancy.

**Methods:**

Observational study that evaluated the gas analysis of the umbilical cord blood collected from a cohort of healthy newborns with gestational age ≥37 weeks. Umbilical venous and arterial oxygen levels as well as fetal oxygen extraction were calculated to establish whether oxygenation level changes over the last weeks of pregnancy. In addition, fetal lactate, and carbon dioxide production were analyzed to establish whether oxygen oscillations may induce metabolic effects *in utero*.

**Results:**

This study demonstrates a progressive increase in fetal oxygenation levels from the 37th to the 41st weeks of gestation (mean venous PaO_2_ approximately from 20 to 25 mmHg; *p* < 0.001). This increase is largely attributable to growing umbilical venous PaO_2_, regardless of delivery modalities. In neonates born by vaginal delivery, the increased oxygen availability is associated with a modest increase in oxygen extraction, while in neonates born by cesarean section, it is associated with reduced lactate production. Independently from the type of delivery, carbon dioxide production moderately increased. These findings suggest a progressive shift from a prevalent anaerobic metabolism (Warburg effect) towards a growing aerobic metabolism.

**Conclusion:**

This study confirms that fetuses grow in a hypoxic environment that becomes progressively less hypoxic in the last weeks of gestation. The increased oxygen availability seems to favor aerobic metabolic shift during the last weeks of intrauterine life; we hypothesize that this environmental change may have implications for fetal maturation during intrauterine life.

## 1. Introduction

Oxygen is thought to play a major role in modulating embryo and fetus growth, although embryonic development occurs under anaerobic conditions and the fetus thrives in a very-low oxygen environment (1). Before the 10th week of human gestation, placental oxygen is less than 20 mmHg (approximately 2% O_2_), similar to the level detectable within the non-pregnant uterus (2, 3). This hypoxic environment affects the metabolic adaptation of the embryo and fetus by stimulating glycolytic metabolism through an increased uptake of glucose, which instead to be used for mitochondrial oxidative phosphorylation, is converted to lactate, similarly to what is observed in cancer cells (the so- called Warburg effect) (4).

The intrauterine low oxygen tension is known to inhibit tissue differentiation (5, 6), but promotes human trophoblast cell proliferation (7) and embryo vascularization through the upregulation of hypoxia-inducible factors that activate a plethora of proangiogenic cytokines (8). Therefore, during the first weeks of pregnancy, low oxygen environment promotes placental development that in turn induces three-fold increase of placenta oxygenation to around 60 mmHg (approximately 8% O_2_) during the second trimester of gestation (2). Oxygen transfer from the mother to the fetus occurs by simple diffusion *via* the placenta. However, several structural obstacles tend to reduce the oxygen-diffusing capacity of the placenta, as demonstrated by the fact that the partial pressure of oxygen (PaO_2_) of the umbilical venous blood, which transfers oxygen from the placenta to the fetus, is significantly lower than maternal arterial PaO_2_ (9). Therefore, the tripled placental oxygen content during the transition from the first to the second trimester of gestation favors an increasing oxygen availability to the feto-placental unit and explains why the environment where the embryo/fetus grows and matures can be considered a dynamic habitat where hypoxia gradually decreases.

Extrapolations from animal studies suggest that human fetal arterial PaO_2_ is approximately 20 mmHg (10), while in humans, umbilical venous PaO_2_ is approximately 28 mmHg during the third trimester of pregnancy (11). Data on fetal oxygenation with advancing gestation suggests a progressive reduction toward the near-term of pregnancy (12–16). However, little is known about the trend of fetal oxygenation during the last weeks of pregnancy and in particular from the thirty-seventh week to term, although, in a recent study, umbilical vein oxygenation has been reported to increase over the last weeks of pregnancy supporting an increased placental transport efficiency for oxygen as a primary determinant of fetal growth (17).

Umbilical-cord blood gas sampling is the most reliable indicator of fetal metabolic condition and arterial/venous cord blood samples can be collected for all births whenever possible (18). In the present study, umbilical-cord blood gas samples have been analyzed in order to establish whether oxygenation level changes during the last weeks of pregnancies in newborns with gestational age (GA) ≥37 weeks. The possibility that changing oxygenation may induce metabolic effects *in utero*, i.e., impacting the Warburg effect, has been also analyzed.

## 2. Methods

The study was conducted in the University Hospital of Pisa, Italy, with approximately 1,700 births per year. All the neonates born between January 1st, 2019, and December 31st, 2019 were enrolled. Their umbilical-cord blood samples were collected at about 60 s after birth following delayed cord clamping. Samples collected from preterm newborns (<37 weeks) or gestations with fetal or maternal intrapartum complications (i.e., fetuses with an abnormal intrapartum cardiotocography that required emergency cesarean section, operative vaginal delivery involving application of forceps or a vacuum extractor, meconium-stained amniotic fluid, cord prolapse, placental abruption, chorioamnionitis, maternal sepsis, hemorrhage, convulsions, uterine rupture, cord avulsion) were excluded from the study. Umbilical-cord blood samples with missing values or suggestive of severe acidosis at birth (pH ≤ 7.00 and/or BE ≤ −12 mmol/L) (19, 20) were excluded from the analysis.

In accordance with recent studies, values of umbilical (venous and arterial) parameters < or >3 SD from their respective means were individually evaluated and (i) corrected if probably mis-entered, (ii) retained unchanged if considered plausible, and (iii) excluded if considered implausible (17). We excluded from the analysis cord blood gas whose results did not fulfill the following criteria: (i) arterial pH < the venous pH (by at least a difference of 0.022) and (ii) arterial partial pressure of carbon dioxide (PaCO_2_) > the venous PaCO_2_ (by at least a difference of 5.3 mmHg) (21). Finally, the remaining umbilical (venous and arterial) PaO_2_, PaCO_2_, pH, and lactate values <0.5th percentile and >99.5th percentile were additionally excluded, to remove extreme values potentially reflective of pronounced intrapartum events (e.g., fetal asphyxia, maternal hyperventilation) (17).

An approximate 20 cm segment of the cord was isolated and cut between a set of two clamps. Cord blood was collected by blood gas syringes containing spray-dried calcium-balanced lithium heparin. First, a minimum of 0.2 ml of blood was withdrawn from the artery, and then a second syringe was used to obtain a venous sample. The samples were labeled and identified as arterial or venous and analyzed as soon as possible after their collection, using an automatic blood gas analyzer (GEM® Premier 4000, Instrumentation Laboratory, Lexington, MA, USA). The pH, PaCO_2_, PaO_2_, and lactate were measured, whereas base excess was calculated using the formula described by Siggaard- Anderson: (1–0.014 × Hb)×[HCO_3_^−^ −24.8 + (1.43 × Hb + 7.7)×(pH−7.4)] (22). Oxygen content was calculated using the formula [(1.34 × Hb × O_2_ saturation/100) + (0.0031 × PaO_2_)]. Fetal oxygen extraction was calculated as the difference between umbilical venous and arterial blood oxygen contents divided by umbilical venous oxygen content. Fetal lactate and CO_2_ productions were calculated, respectively, as the difference between arterial lactate or blood CO_2_ and venous contents, divided by the respective venous content.

Institutional protocols for pain and anesthesia management included epidural analgesia or anesthesia for all healthy parturient women and performed by ropivacaine or levobupivacaine in combination with sufentanil. General anesthesia was indicated for emergency cesarean sections. Maternal oxygen supplementation was reserved for the management of abnormal fetal heart rate tracings only.

### 2.1. Statistical analysis

Categorical data were described by absolute and relative (%) frequency, and continuous data were summarized by means and standard deviations. To compare delivery type with categorical and continuous factors, chi-square test and *t*-test for independent samples were performed, respectively. Furthermore, GA was compared with several outcomes such as venous or arterial PaO_2_, oxygen extraction, venous or arterial lactate, lactate production, venous or arterial PaCO_2_, and PaCO_2_ production by simple linear regression, and the beta coefficient was also calculated. A *p* < 0.05 was considered significant. Statistical analysis was done with SPSS 28.0 program (IBM SPSS Statistics for Windows, Version 28.0 Armonk, NY: IBM Corp.).

### 2.2. Ethical approval

The present study was performed following the ethical principles for medical research involving human subjects adopted by the Word Medical Association General Assembly (Declaration of Helsinki) and its later amendments. The study was approved by the Pediatric Ethical Committee for Clinical Research of Tuscany region (number 291/2022).

## 3. Results

**Figure 1:**
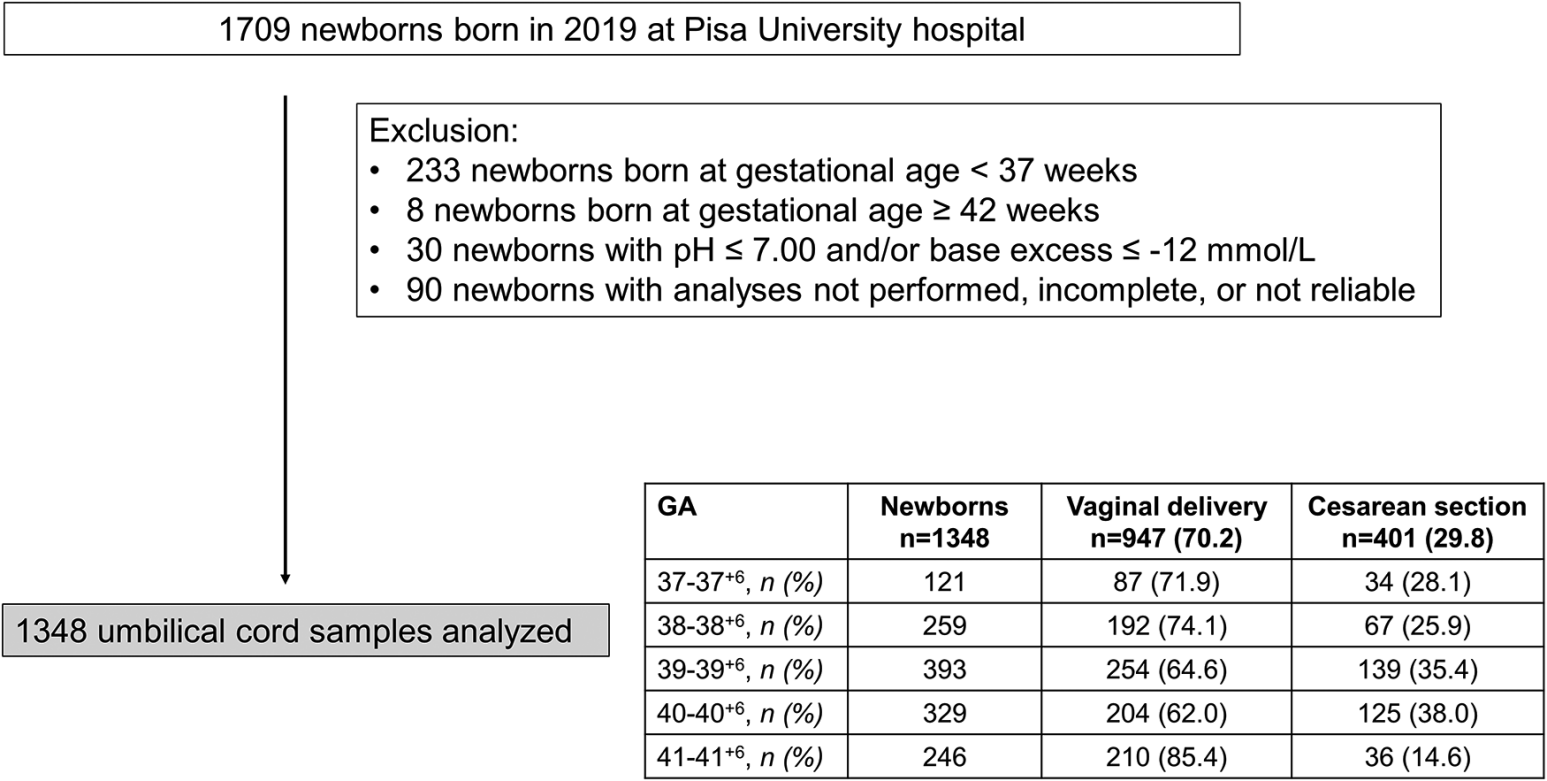
Flow chart illustrating patient enrollment of this retrospective observational cohort study.

Out of 1,709 neonates born in 2019, 233 newborns with GA <37 weeks and 8 with GA ≥ 42 were excluded from the analysis. Thirty patients with acidosis at birth, and 90 patients in which umbilical-cord gas analyses were not performed, grossly incomplete, or unreliable were excluded. Of 1,348 newborns enrolled, females were prevalent (700/1,348, 51.9%). 947 newborns were born from vaginal delivery (70.2%), but this percentage widely varied during the study period (Figure 1).

**Figure 2:**
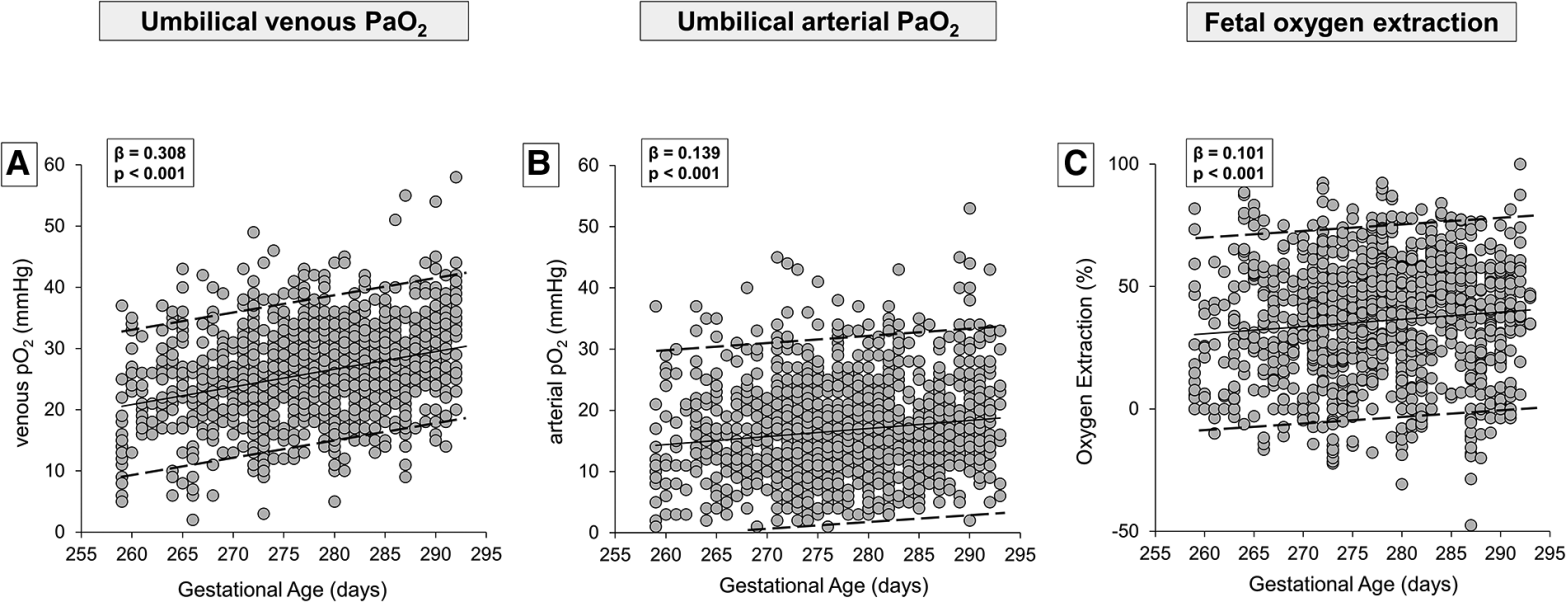
Scatter plots with regression lines and 95% prediction intervals representing umbilical-cord oxygenation status [venous PaO_2_, panel **A** (*n* = 1,233); arterial PaO_2_, panel **B** (*n* = 1,181); fetal oxygen extraction, panel **C** (*n* = 1,158)] of the whole study population.

In Table 1, the demographic and gas analytical parameters of all newborns are shown. Neonates born by spontaneous delivery showed higher values of PaO_2_ and lactate, and lower values of PaCO_2_ and base excess both in venous and arterial cord samplings. Neonates born by cesarean section exhibited an oxygen extraction significantly higher than neonates born by spontaneous delivery. Newborns showed comparable Apgar scores throughout the analyzed period, regardless of GA progression (Supplementary Figure S1). To evaluate whether oxygen tension changed with the progression of pregnancy, data of venous and arterial PaO_2_ as well as oxygen extraction were evaluated in function to the progression of pregnancy (Figure 2). Values from umbilical venous samples showed a progressive, linear increase in PaO_2_ levels from 37 to 41 weeks (on average an increase of about 1 mmHg per week) (Figure 2A), while arterial PaO_2_ increased with a lower slope (Figure 2B). Overall, oxygen extraction slightly increased from 37 to 41 weeks, in parallel with an increased oxygen availability (Figure 2C). Supplementary Table S1 shows numerical data stratified by weeks of gestation.

**Table 1:** Umbilical-cord blood gas analysis in all enrolled term newborns and separately analyzed by the type of delivery.

	All term newborns *n* = 1,348	Vaginal delivery *n* = 947	Cesarean section *n* = 401	*p* value
GA, weeks, mean (SD)	39.7 (1.2)	39.7 (1.2)	39.6 (1.1)	0.169
Birth weight, g, mean (SD)	3,334 (978)	3,370 (1,125)	3,248 (462)	0.036
Male, *n* (%)	648 (48.1)	465 (49.1)	183 (45.6)	0.169
Apgar score at 5 min, mean (SD)	8.9 (0.5)	9.0 (0.4)	8.9 (0.5)	0.507
**Umbilical venous cord sampling**				
pH, mean (SD)	7.318 (0.07)	7.319 (0.07)	7.315 (0.06)	0.304
PaCO_2_, mmHg, mean (SD)	39.9 (8.3)	38.4 (8.3)	43.6 (7.5)	<0.001
PaO_2_, mmHg, mean (SD)	25.9 (7.7)	27.4 (7.5)	22.8 (7.3)	<0.001
BE(B), mmol/L, mean (SD)	−5.4 (3.1)	−6.0 (2.9)	−4.0 (3.2)	<0.001
Lactate, mmol/L, mean (SD)	3.6 (1.5)	4.0 (1.5)	2.3 (1.3)	<0.001
**Umbilical arterial cord sampling**				
pH, mean (SD)	7.238 (0.089)	7.232 (0.07)	7.246 (0.07)	<0.001
PaCO_2_, mmHg, mean (SD)	52.4 (11.4)	52.0 (11.8)	53.4 (10.8)	0.045
PaO_2_, mmHg, mean (SD)	16.7 (7.8)	18.9 (7.6)	12.0 (6.0)	<0.001
BE(B), mmol/L, mean (SD)	−5.9 (3.6)	−6.5 (3.1)	−4.7 (4.3)	<0.001
Lactate, mmol/L, mean (SD)	4.0 (1.6)	4.4 (1.6)	2.7 (1.4)	<0.001
Veno-arterial O_2_ difference, mmHg, mean (SD)	9.5 (7.1)	8.8 (7.3)	11.1 (5.7)	<0.001
Fetal oxygen extraction, %, mean (SD)	35.9 (24.3)	30.3 (24.1)	48.0 (19.8)	<0.001

GA, gestational age; PaO_2_, partial pressure of oxygen; PaCO_2_, partial pressure of carbon dioxide; BE, base excess.

The type of delivery was demonstrated to influence significantly the gas analysis results, mostly oxygenation (Table 1). To assess whether this influence was due to labor itself, data on neonates born by cesarean section were disaggregated according to the presence of labor. Neonates born with previous labor were 83 (20.7%), and without labor were 318 (79.3%). The results showed no significant differences between the newborns without or with labor: mean venous PaO_2_ was 22.4 ± 7.2 mmHg in neonates born without labor vs. 24.2 ± 7.2 mmHg in neonates born after labor (*p* = 0.065), and oxygen extraction was 48.2 ± 24.6% vs. 47.5 ± 22.8% (*p* = 0.553). There was no statistical difference between the newborns without or with labor either regarding venous PaCO_2_ (43.8 ± 7.9 vs. 42.9 ± 6.4 mmHg; *p* = 0.302), arterial PaCO_2_ (53.5 ± 11.1 vs. 52.8 ± 9.3 mmHg; *p* = 0.632), venous base excess (−4.0 ± 3.2 vs. −4.3 ± 2.9 mmHg; *p* = 0.430), or arterial base excess (−4.7 ± 4.4 vs. −4.6 ± 3.8 mmHg; *p* = 0.745). Conversely, mean venous lactate was significantly lower in neonates born without labor (2.2 ± 1.1 vs. 2.6 ± 1.6 mmol/L; *p* = 0.047), while mean arterial lactate (2.6 ± 1.3 vs. 2.9 ± 1.5 mmol/L; *p* = 0.109) did not reach statistical significance.

**Figure 3:**
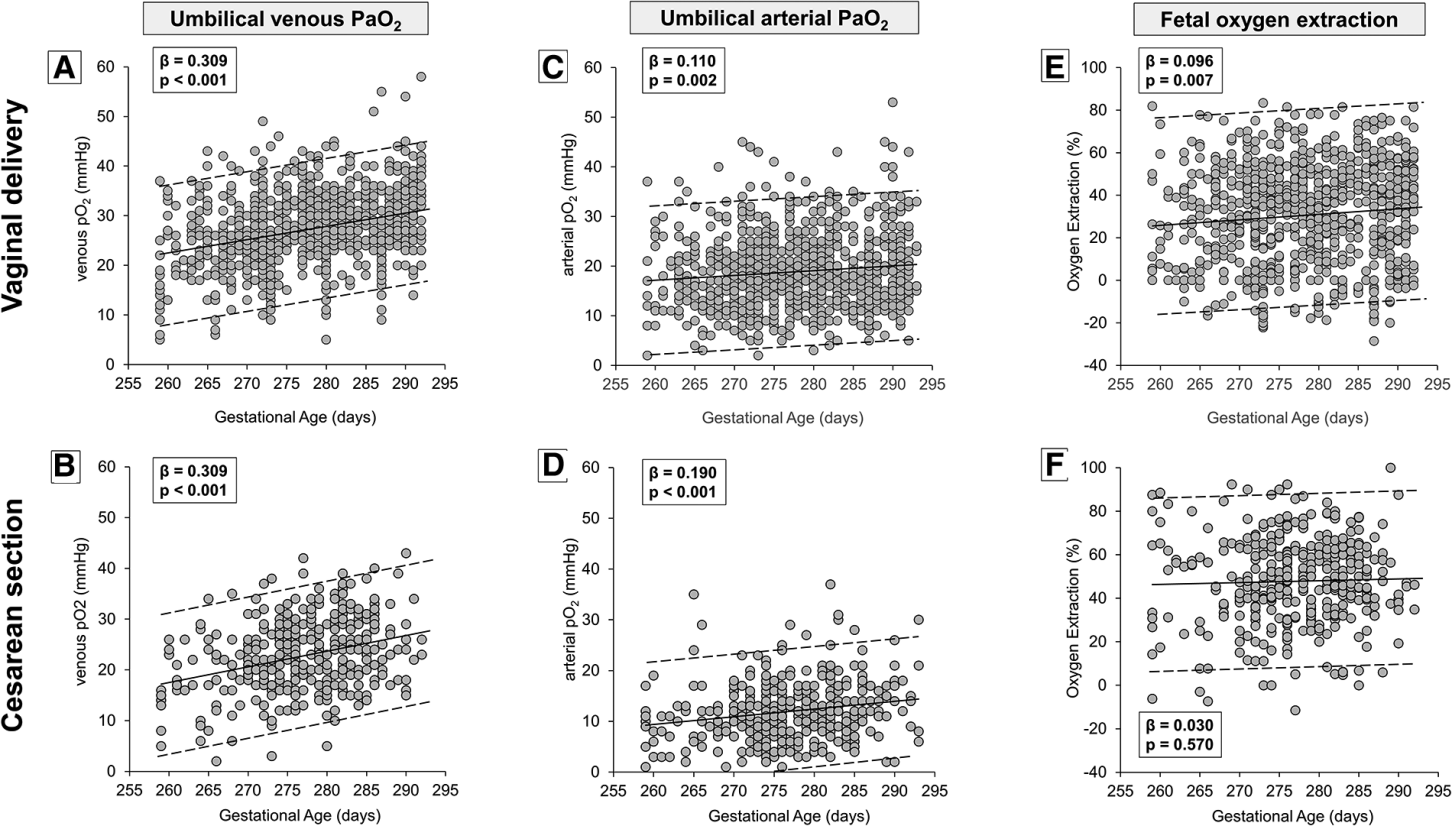
Scatter plots with regression lines and 95% prediction intervals representing umbilical-cord oxygenation status [venous PaO_2_, panel **A** (*n* = 860)—panel **B** (*n* = 373); arterial PaO_2_, panel **C** (*n* = 811)—panel **D** (*n* = 370); fetal oxygen extraction, panel **E** (*n* = 795)—panel **F** (*n* = 363)] stratified by modality of delivery.

Therefore, umbilical-cord oxygen values (venous, arterial, and oxygen extraction) were analyzed separately according to the type of delivery (Figure 3). Results confirmed a progressive and linear increase in umbilical venous PaO_2_ from the 37th to the 41st week, both in neonates born from vaginal delivery (Figure 3A) and cesarean section (Figure 3B). The increase of arterial PaO_2_ was less striking if compared with venous PaO_2_ (Figures 3C,D). Overall, the increase in oxygen extraction was modest and significant only in neonates born by vaginal delivery (Figures 3E,F). Numerical data stratified by weeks of gestation are shown in Supplementary Table S2.

**Figure 4:**
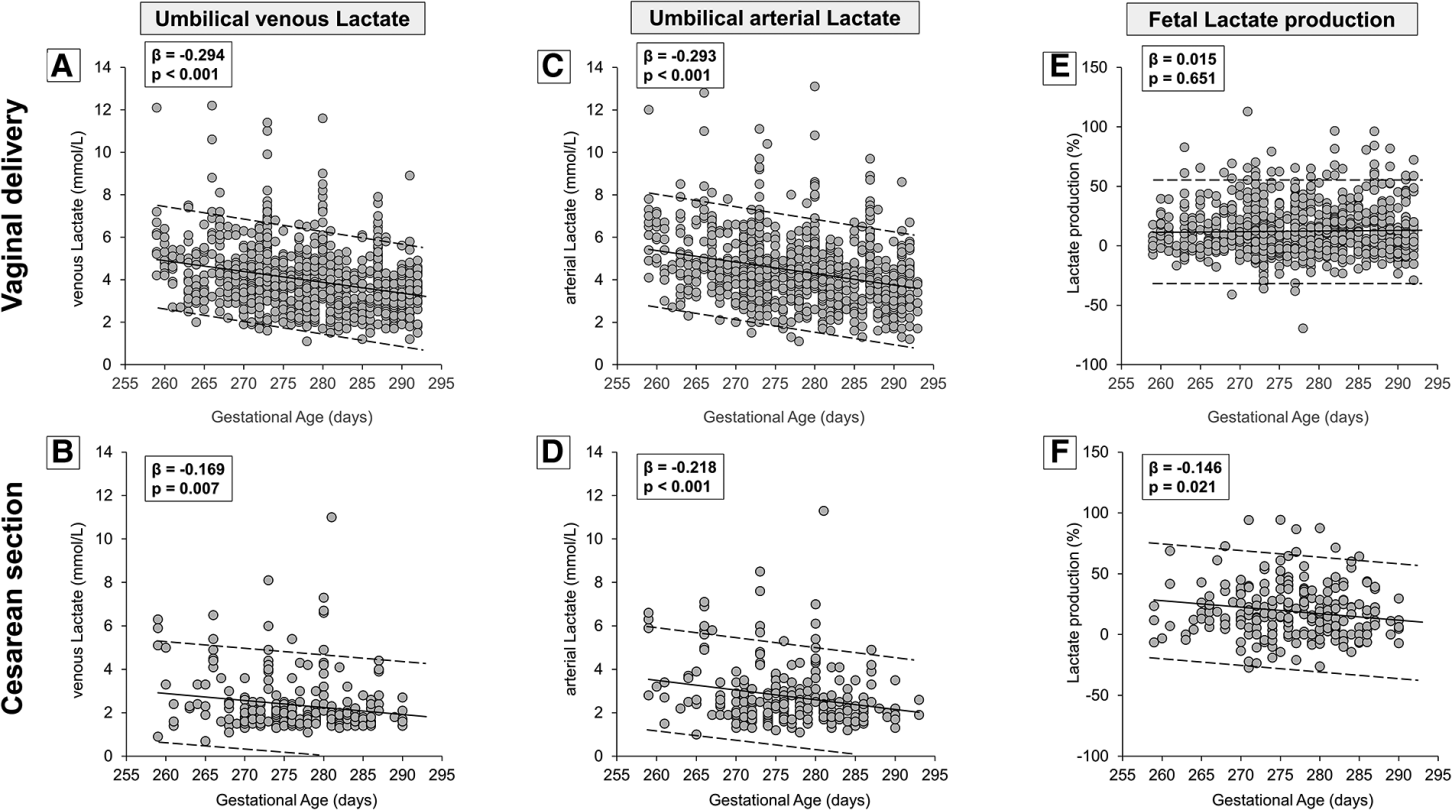
Scatter plots with regression lines and 95% prediction intervals representing umbilical-cord lactate levels [venous lactate, panel **A** (*n* = 893)—panel **B** (*n* = 284); arterial lactate, panel **C** (*n* = 909)—panel **D** (*n* = 283); fetal lactate production, panel **E** (*n* = 889)—panel **F** (*n* = 278)] stratified by modality of delivery.

The modality of delivery influenced significantly also the values of lactate. Newborns born by vaginal delivery exhibited higher values of lactate both in venous and arterial samples (Table 1). To evaluate whether lactate level was also affected by the progression of GA, results were analyzed separately according to delivery modality. Both in neonates born by vaginal delivery and by cesarean section a progressive, linear decrease in lactate levels was observed, either in venous (Figures 4A,B) or in arterial samples (Figures 4C,D), from the 37th to the 41st week. Fetal lactate production seemed to be stable throughout the period considered in neonates born by vaginal delivery, (Figure 4E), but decreased in neonates born by cesarean section (Figure 4F). Numerical data stratified by weeks of gestation are shown in Supplementary Table S2.

**Figure 5:**
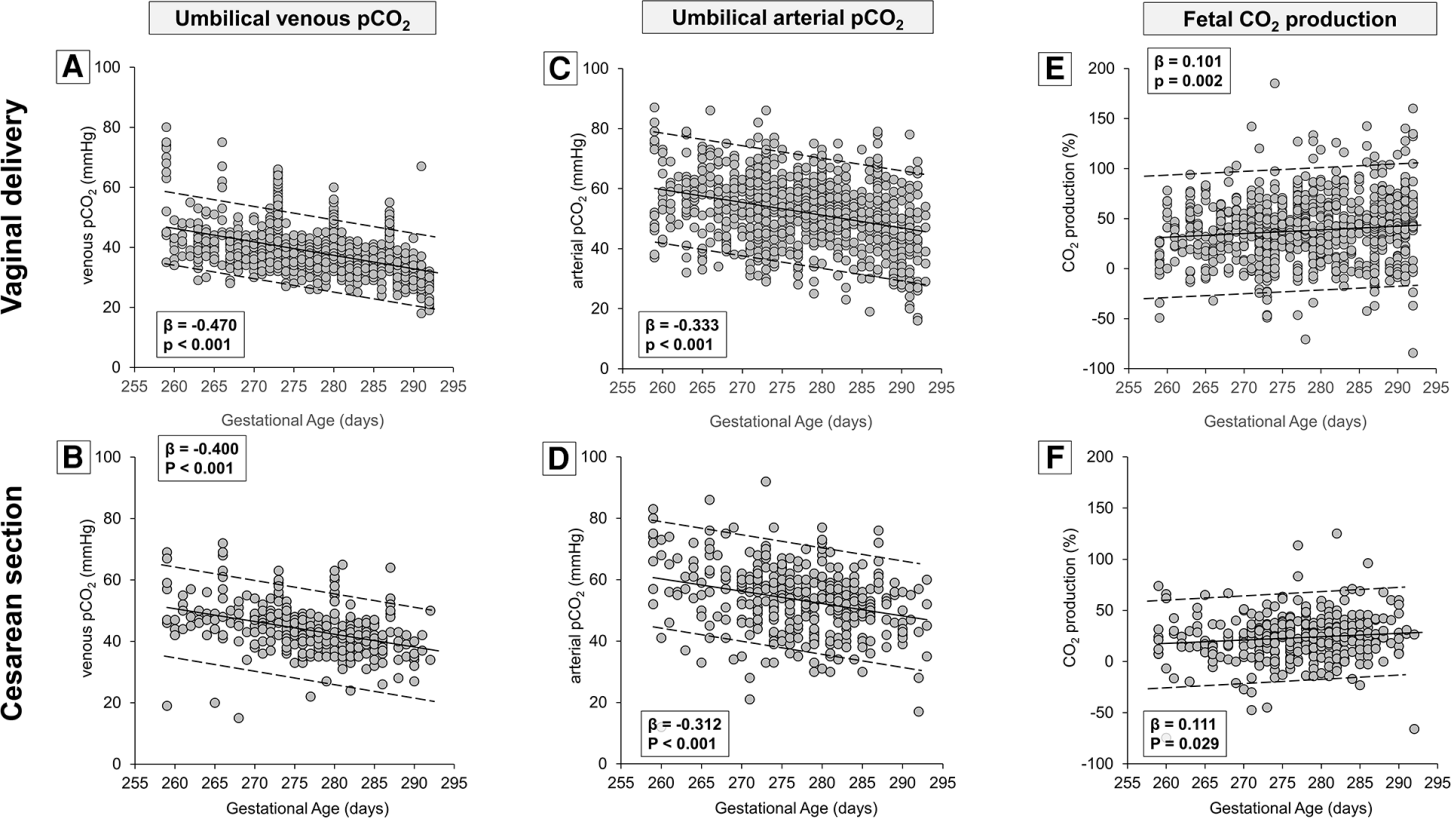
Scatter plots with regression lines and 95% prediction intervals representing umbilical-cord carbon dioxide levels [venous PaCO_2_, panel **A** (*n* = 934)—panel **B** (*n* = 388); arterial PaCO_2_, panel **C** (*n* = 943)—panel **D** (*n* = 396); fetal CO_2_ production, panel **E** (*n* = 932)—panel **F** (*n* = 385)] stratified by modality of delivery.

Table 1 demonstrates that the type of delivery affected also venous PaCO_2_ levels, suggesting the opportunity to analyze CO_2_ production separately in newborns born by vaginal delivery or cesarean section. The results demonstrated that the umbilical-cord content of CO_2_ followed an opposing trend if compared with the trend of oxygen, with a linear progressive decrease both in venous (Figures 5A,B) and arterial samples (Figures 5C,D). However, the CO_2_ production seemed to increase progressively per gestation progression independently from the type of delivery (Figures 5E,F). Again, numerical data stratified by weeks of gestation are shown in Supplementary Table S2.

## 4. Discussion

It is broadly accepted that physiologic hypoxia plays an important role in embryonic development (23). In hypoxic environment, before placenta development, embryonic stem cells maintain their pluripotency and hypoxia promotes their proliferation (24). In fact, cytotrophoblast proliferation requires a very-low oxygen tension to maintain an undifferentiated state (25). However, a series of considerations suggest that physiological intrauterine hypoxia is dynamic. In particular, the progressive remodeling of spiral arteries in which maternal vascular smooth muscle cells and endothelial cells are replaced by embryonic extravillous trophoblasts (26) allows the establishment of the placental circulatory system, which permits an increased oxygen delivery to the rapidly growing fetus (27). The chick embryo model makes it possible to directly study the effects of varying fetal oxygenation levels on prenatal growth, demonstrating the role of oxygen as a developmental morphogen. Indeed, an increased oxygen availability increases the growth of the chick (28–30). This is indicative of the possibility that the progressive increase in oxygen availability ensured by the complete development of the placenta is functional to the growth and maturation of the embryo/fetus during the first trimester of pregnancy. While in the subsequent trimesters, a pathological reduction in fetal oxygenation leads to restricted fetal growth and well-being (31), it is not clear whether variations in environmental oxygenation participate to the physiological fetal growth.

Previous findings from the analysis of human umbilical-cord arterial and venous blood samples obtained *via* cordocentesis demonstrate a decreased fetal PaO_2_ and O_2_ saturation between second and third trimester of gestation (12–16) thus in apparent contradiction with the present results demonstrating instead an increased oxygenation during the last weeks of gestation, as also shown by Richardson et al. (17). However, the comparison of such results is questionable because, on the one hand, studies based on cordocentesis depict a more physiological state over a wide GA range before the peripartum period but did not take into account the last weeks of pregnancy; on the other hand, these studies were obtained from pregnancies complicated by maternal infections, fetal structural anomalies, or various prenatal pathologies (12–16). Although studies in healthy fetal animals report no significant changes in blood gas and acid-base variables during gestation (32–39), their findings are difficult to be translated to human intrauterine gestation. For example, sheep maintain the same umbilical gas status throughout the latter half of pregnancy due to their high umbilical flow rate, while human fetuses rely on increased oxygen extraction or more efficient placental diffusion capacity as pregnancy progresses (40). In addition, in many animals including rodents, several anatomic structures are still immature at birth (41–43) and their maturation is associated, at least chronologically, with an increase in oxygen exposure after birth. This is particularly evident in the retina, where the superficial vascular plexus reaches its maturation postnatally during the first week after birth (42) or even later (44), thus suggesting that an increased oxygen tension may be determinant in the extrauterine maturation process. In contrast, fetal human retinas complete vascularization during the final weeks of intrauterine life (45), suggesting the progressive increase in oxygen tension during this time may contribute to this process.

To determine whether the oxygenation status changes over the last weeks of pregnancy requires the analysis of a large series of umbilical-cord samples that represents a safe, non-invasive approach to obtaining suggestive information (46), even though they were exclusively obtained during the peripartum period. Despite this evident limitation, the simplicity of the method, its low cost, and its non-invasiveness, made it possible to have a high number of examinations available to assess the state of fetal oxygenation.

As shown by the present results (Figure 2), fetal oxygenation linearly and progressively increases from the 37th to the 41st week of gestation. This finding, however, could be questioned by the modality of delivery as the oxygen content measured in neonates born by cesarean section is significantly lower than in neonates born by vaginal delivery (Table 1). To verify if the fetal oxygenation level increases with gestation independently from the type of delivery, umbilical-cord gas analytic data have been analyzed separately between neonates born from vaginal delivery or cesarean section (Figure 3). The data, despite their wide variability, confirm an increase of venous PaO_2_ availability as GA increases, independently of delivery modality. This increase is progressive but slight, statistically appreciable only thanks to the large sample size. The effect of the delivery modality on fetal oxygenation does not seem to be dependent on the occurrence of labor as in neonates born by cesarean section, the effect of labor on the PaO_2_ level does not reach statistical significance. However, this result might depend on the sample size, and therefore this aspect deserves further analysis in future studies. On the contrary, the different fetal oxygenation depending on the delivery modality may be related, regardless of the clinical conditions that recommended cesarean section, to a disparity in the placental flow. In fact, compared to vaginal delivery, cesarean section is usually characterized by a lower umbilical transfusion force because of the position of the newborns on the mother's abdomen with a gravity gradient that may prevent the cord blood flow from the placenta to the newborn (47). In addition, maternal hypotension (48) or reduced uterine contractile force that are common among women subjected to cesarean sections due to uterine incision and anesthesia (49, 50) may explain the higher oxygen extraction possibly due to the need to guarantee fetuses with comparable oxygen availability. The oxygen extraction calculated in neonates born by vaginal delivery (around 30%) is very similar to the value recently measured by MRI in human fetuses *in utero* at 36 ± 1 weeks of GA (51), indicating that the blood gas measurements performed in neonates born by vaginal delivery may be more representative of intrauterine conditions. The reduced umbilical flow due to the fetus position above the placenta might also limit the diffusion of CO_2_ from the fetus to the mother, explaining the higher PaCO_2_ values in neonates born by cesarean delivery. In addition, the modality of delivery also significantly influences the values of lactate, as demonstrated by its higher concentration in neonates born by vaginal delivery. This increased concentration might be related to the contribution of the lactate produced by the fetus during the second stage of labor (52). This is also suggested by the present study demonstrating higher values of lactate in neonates born by cesarean section with previous labor.

As shown by the present results, between the 37th and the 41st week, a progressive increase in oxygen availability coincides with a progressive reduction of lactate (Figure 4) and CO_2_ delivery (Figure 5) from the placenta to the fetus, associated with an increased fetal CO_2_ production and a reduced lactate production, which was only observed in neonates born by cesarean section likely due to the lack of lactate production during fetal expulsion. Therefore, the reduced lactate production found in cesarean births may provide a picture more representative of the intrauterine condition and could be potentially generalized to all fetuses over the last weeks of pregnancy. These findings suggest for fetuses at term a progressive and linear shift from a metabolism predominantly anaerobic, typical of intrauterine embryonic life (Warburg effect) and characterized by a considerable extrusion outside the cells of lactate (4, 53), toward a growing aerobic metabolism (54).

The increase of oxygen availability to fetuses during the last weeks of gestation is probably more relevant than that estimated here because in late gestation the hemoglobin concentration is higher compared to early or mid-pregnancy (55), and the percentage of fetal hemoglobin molecules decreases as adult hemoglobin (which has lower affinity for oxygen) begins to rise (56). The fact that oxygen extraction is only slightly increased irrespectively of the increased oxygen availability is in line with previous findings (17). In this respect, the disproportion between the increased oxygen availability and the modest increase in fetal extraction suggests that growing oxygenation would not be able to ensure greater oxygen consumption but would rather represent a signal capable of inducing cellular metabolic shift. In this line, an MRI study has recently demonstrated that fetal oxygen consumption remains substantially unchanged between 33 and 38 weeks despite increased oxygen extraction (40).

Although much work has been focused on the role of hypoxia in maintaining the stemness traits or in promoting dedifferentiation, the possibility that maturation or differentiation is driven by increasing concentrations of oxygen has been less emphasized (1, 57–59). As shown by the present findings, slight but progressive increase in fetal oxygenation, largely attributable to an increased oxygen transfer from the placenta to the fetus, occurs over the last weeks of pregnancy presumably due to structural changes of the aging placenta that favors an increase of the oxygen diffusing capacity (60).

Although it is not possible to attribute with certainty a biological role to this progressive increase in oxygenation over the last weeks of pregnancy, the idea that oxygen modulates cellular differentiation is widely accepted, even if tissue-specific (58). In some anatomic zones, as in the rat central nervous system, hypoxia promotes the differentiation of stem cells into differentiated cells (61), in other districts including the neuroretina (62), pancreatic β-cells (63), or keratinocytes, stem cell differentiation is triggered by increased oxygenation (64). Therefore, it is reasonable to speculate that the intrauterine environment, which physiologically becomes more hypoxic from mid-gestation to near-term and then less hypoxic until term, ensures a tissue-specific fetal maturation and cellular differentiation depending on oxygen tension. In the retina, for instance, hypoxia induces astrocytes to differentiate, to stop their migration, and to produce VEGF (65), while the differentiation of endothelial cells requires a more oxygenated environment (59). This unlike differentiation induced by different oxygen concentrations might explain why some organs, such as the retina, vascularize after birth in rodents while within the uterus in humans.

Among the myriad of mechanisms that can be activated by oxygen dynamics during intrauterine life, oxygen-sensing mechanisms induced by catecholamines have been suggested to actively participate in the fetal growth (17). In the β-adrenoceptor (β-AR) family, recent studies have demonstrated a close relationship between oxygen levels and the expression of its last cloned receptor, the β3-AR (66). Under hypoxic conditions, β3-AR is significantly up-regulated in human pregnant myometrium where it is the predominant β-AR subtype that contributes to inhibiting spontaneous contractions (67, 68), as confirmed by the induction of preterm delivery after the administration of β3-AR antagonists in pregnant mice (69). Interestingly, β3-AR is actively involved in cancer and embryonic stem cell metabolic programming (70), differentiation (71), and in the induction of fetal immune tolerance (72), suggesting the idea that this receptor plays an important role during fetal development. In the meantime, recent studies have demonstrated that β3-AR is rapidly down-regulated upon exposure to a more oxygenated environment (73) confirming the strict dependence of β3-AR expression on oxygen levels. The relevant functional role of β3-AR during fetal life together with its close correlation with oxygen levels explains why it is important to know how oxygen levels change during physiological pregnancies, and at the same time suggests the possibility that its precocious down-regulation after premature birth might affect the newborn health (74).

Finally, the finding that oxygen levels vary according to gestational age has important implications for the emerging artificial placenta technology which is currently being developed and being tested on animals (75). In this regard, it would be important to know what oxygenation levels (and other gas parameters) need to be reached at each point in gestation to support normal physiologic growth and maturation of the fetus while in the artificial placenta technology.

### 4.1. Limitations

The main limitation of the present study is the use of umbilical-cord samplings as indirect indicators of the real intrauterine condition. While gas analytic values after vaginal delivery are affected by fetus engagement through the birth canal and the reduced blood flow during uterine contractions (76), values after cesarean section are conditioned by non-physiological management including cardiovascular effects of anesthesia, maternal ventilation, and maternal position (77, 78). Beside sampling or test execution errors, gas values can be influenced by hypoxic-ischemic suffering during labor, the effects of which may depend on labor duration (79, 80). In addition, umbilical-cord sampling does not include additional parameters such as the umbilical blood flow rate, which plays a key role in oxygen delivery (81), thus we assumed that umbilical flow remained stable or increased over the last weeks of gestation.

In this study, the umbilical-cord blood samples have been collected approximately 60 s after birth. Overall, this procedure does not significantly affect the cord blood acid-base and gas values, even though samples collected after delayed cord clamping show arterial PaO_2_ values approximately 1 mmHg lower than samples collected immediately after delivery (early cord clamping) (82). Such difference is not negligible, because it roughly corresponds to the variations in oxygenation measured weekly in our study. Although additional data from premature newborns would have provided a better understanding of the dynamics of fetal oxygenation, the pooling of data obtained from term newborns with those from preterm newborns would not have been methodologically correct, as umbilical-cord blood samples in neonates born prematurely are usually performed by blood collected after early cord clamping. However, other studies performed on a homogeneous cohort of preterm infants are needed to evaluate the oxygenation trend before the 37th week because the results of the present study are apparently conflicting with previous studies, performed in fetuses from mid-gestation to near-term, in which a progressive reduction of fetal oxygenation has been reported (12–16), suggesting a biphasic trend of oxygenation during intrauterine life.

The fact that the oxygenation in the blood collected after delayed cord clamping is lower than in samples collected immediately after delivery (82) reduces the possibility that spontaneous respiration may have played a role in umbilical gas status. Consequently, also the hypothesis that the increased PaO_2_ and the reduced PaCO_2_ observed in newborns with higher GA might be influenced by a different lung maturity or respiratory strength seems unlikely, as confirmed also by the comparable Apgar scores regardless of GA progression (Supplementary Figure S1).

## 5. Conclusion

This study confirms that in the last weeks of gestation, intrauterine hypoxia is dynamic and therefore potentially capable of modulating differentiation processes. Understanding the physiological dynamics of oxygen during intrauterine life assumes even greater importance now that mechanisms, which link oxygen-associated transcription factors to their transactivated proteins are available: this means that the way to pharmacologically mimic the effects of oxygen oscillations is open. More detailed knowledge of the fluctuations in oxygen levels starting from conception and throughout pregnancy, their biological effects during intrauterine life, and the mechanisms through which oxygen regulates cell differentiation might help to identify specific time windows on which to intervene to reproduce the effects of oxygen oscillations when, for example, the pregnancy results in a premature birth. Such a perspective would restore the benefits of intrauterine life from which premature infants have been deprived (74).

## Data Availability

The raw data supporting the conclusions of this article will be made available by the authors, without undue reservation.
