# Supplementary material for: Fetal oxygenation in the last weeks of pregnancy evaluated through the umbilical cord blood gas analysis
Source: Front Pediatr. 2023 Apr 21;11:1140021. doi: 10.3389/fped.2023.1140021 (PMC10160648; doi:10.3389/fped.2023.1140021)
Supplement: Supplementary file 1 [file Datasheet1.pdf]

Supplementary Table 1. Umbilical-cord oxygenation status of the whole study population stratified by gestational ages (weeks of gestation).

weeks	$37\text{-}37^{+6}$	$38\text{-}38^{+6}$	$39\text{-}39^{+6}$	$40\text{-}40^{+6}$	$41\text{-}41^{+6}$
UV PaO_2 mmHg, *mean (SD)*	22.4 (8.3)	24.0 (7.2)	25.6 (7.3)	26.8 (7.3)	28.9 (7.9)
UA PaO_2 mmHg, *mean (SD)*	15.9 (8.6)	16.2 (7.6)	16.1 (7.5)	16.3 (7.4)	19.3 (8.3)
Fetal oxygen extraction, %, *mean (SD)*	31.4 (26.6)	34.1 (22.7)	37.2 (24.7)	39.2 (23.2)	32.8 (25.2)
UV= umbilical venous; UA=umbilical arterial.					
