# Supplementary material for: Fetal oxygenation in the last weeks of pregnancy evaluated through the umbilical cord blood gas analysis
Source: Front Pediatr. 2023 Apr 21;11:1140021. doi: 10.3389/fped.2023.1140021 (PMC10160648; doi:10.3389/fped.2023.1140021)
Supplement: Supplementary file 2 [file Datasheet2.pdf]

Supplementary Table 2. Umbilical-cord oxygenation status of the whole study population stratified by gestational ages (weeks of gestation) and modality of delivery.

Vaginal delivery					
weeks	$37\text{-}37^{+6}$	$38\text{-}38^{+6}$	$39\text{-}39^{+6}$	$40\text{-}40^{+6}$	$41\text{-}41^{+6}$
UV PaO_2 mmHg, *mean (SD)*	24.1 (8.1)	25.2 (7.1)	27.2 (7.2)	28.2 (6.9)	29.6 (7.9)
UA PaO_2 mmHg, *mean (SD)*	18.6 (7.7)	17.8 (7.6)	19.0 (7.3)	18.5 (7.3)	20.3 (8.2)
Fetal oxygen extraction, %, *mean (SD)*	24.9 (23.6)	30.2 (22.2)	29.6 (24.7)	32.8 (24.0)	30.8 (25.2)
UV Lactate, mmol/L, *mean (SD)*	4.6 (1.5)	4.3 (1.5)	4.1 (1.5)	3.8 (1.5)	3.6 (1.3)
UA Lactate, mmol/L, *mean (SD)*	5.2 (1.6)	4.7 (1.5)	4.5 (1.6)	4.2 (1.6)	4.0 (1.5)
Lactate Production %, *mean (SD)*	13.4 (18.3)	13.1 (19.8)	10.8 (23.7)	12.9 (19.2)	13.0 (22.4)
UV $PaCO_2$ mmHg, *mean (SD)*	43.7 (9.0)	40.3 (6.5)	39.3 (7.8)	36.8 (6.3)	33.5 (7.5)
UA $PaCO_2$ mmHg, *mean (SD)*	57.8 (12.0)	55.1 (10.5)	53.4 (10.6)	51.4 (9.9)	47.0 (12.7)
CO_2 Production %, *mean (SD)*	34.4 (27.4)	38.2 (25.8)	39.4 (33.4)	43.7 (44.7)	45.9 (33.0)
Cesarean section					
UV PaO_2 mmHg, *mean (SD)*	18.0 (7.3)	20.8 (6.7)	22.8 (6.7)	24.6 (7.6)	24.5 (6.7)
UA PaO_2 mmHg, *mean (SD)*	9.9 (7.3)	11.6 (5.4)	11.6 (5.4)	12.7 (6.3)	13.6 (6.3)
Fetal oxygen extraction, %, *mean (SD)*	47.0 (27.0)	44.7 (20.9)	49.1 (19.3)	48.9 (18.0)	47.7 (20.2)
UV Lactate, mmol/L, *mean (SD)*	3.0 (1.7)	2.3 (1.1)	2.2 (1.1)	2.4 (1.6)	2.5 (1.0)
UA Lactate, mmol/L, *mean (SD)*	3.3 (1.6)	2.8 (1.5)	2.7 (1.3)	2.7 (1.6)	2.8 (1.2)
Lactate Production %, *mean (SD)*	29.2 (52.5)	19.3 (21.8)	20.4 (22.8)	15.8 (20.9)	14.9 (14.1)
UV $PaCO_2$ mmHg, *mean (SD)*	48.2 (10.5)	47.0 (7.8)	43.2 (5.9)	41.5 (6.6)	40.1 (8.2)
UA $PaCO_2$ mmHg, *mean (SD)*	58.4 (14.5)	56.7 (10.3)	53.8 (9.2)	52.2 (8.9)	51.1 (10.4)
CO_2 Production %, *mean (SD)*	26.8 (34.4)	25.0 (24.2)	26.5 (16.9)	27.7 (19.4)	29.2 (17.8)
UV= umbilical venous; UA=umbilical arterial.					
