# Supplementary figures and images for: Fetal oxygenation in the last weeks of pregnancy evaluated through the umbilical cord blood gas analysis
Source: Front Pediatr. 2023 Apr 21;11:1140021. doi: 10.3389/fped.2023.1140021 (PMC10160648; doi:10.3389/fped.2023.1140021)

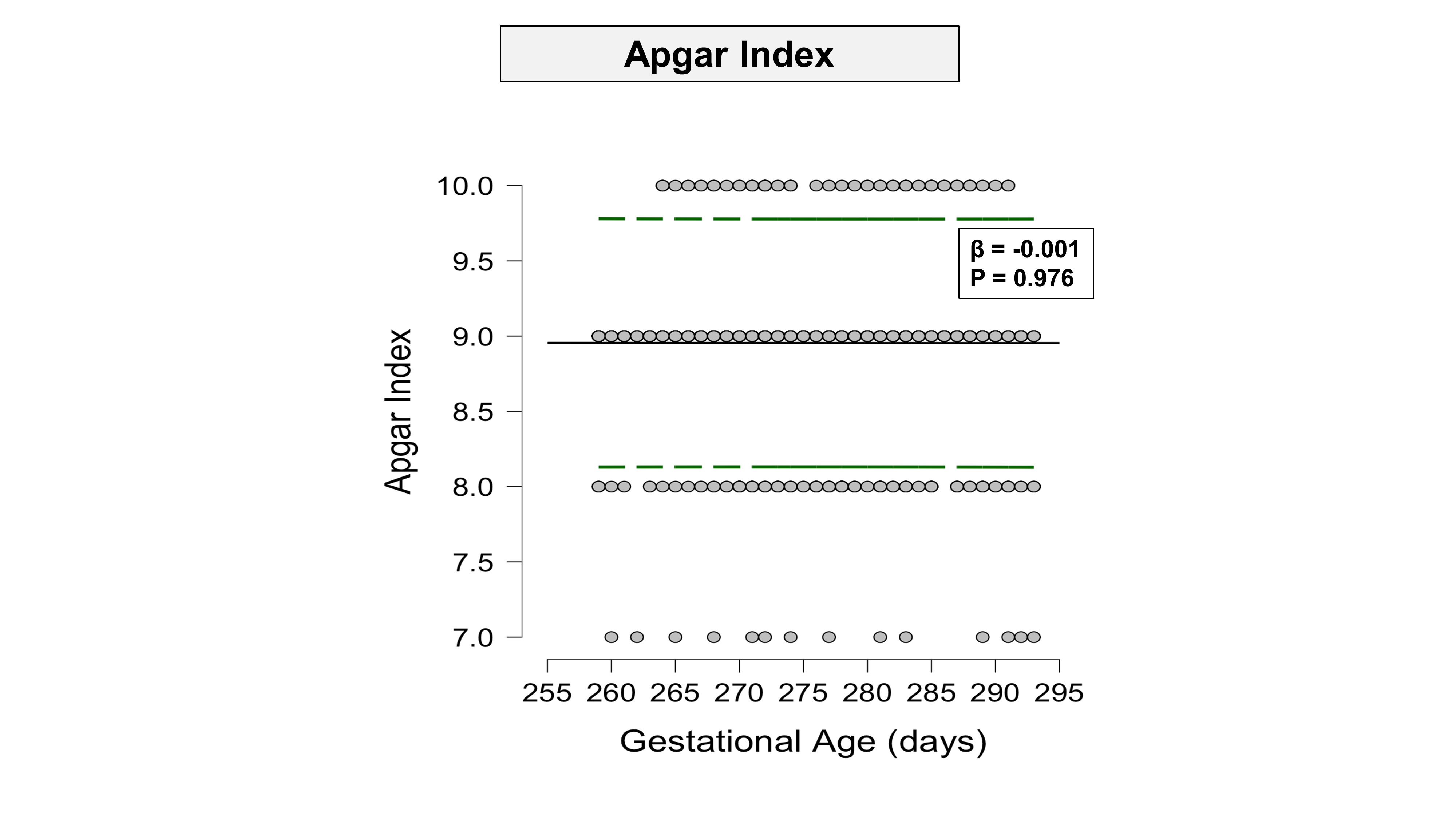

Supplement: Supplementary file 3 [file Image1.tif]
